# Targeting the RAS/MAPK pathway with *miR-181a* in acute myeloid leukemia

**DOI:** 10.18632/oncotarget.11150

**Published:** 2016-08-09

**Authors:** Xiaomeng Huang, Sebastian Schwind, Ramasamy Santhanam, Ann-Kathrin Eisfeld, Chi-ling Chiang, Malori Lankenau, Bo Yu, Pia Hoellerbauer, Yan Jin, Somayeh S. Tarighat, Jihane Khalife, Alison Walker, Danilo Perrotti, Clara D. Bloomfield, Hongyan Wang, Robert J. Lee, Ly James Lee, Guido Marcucci

**Affiliations:** ^1^ Molecular Cellular and Developmental Biology, The Ohio State University, Columbus, OH, USA; ^2^ Nanoscale Science and Engineering Center for Affordable Nanoengineering of Polymeric Biomedical Devices, The Ohio State University, Columbus, OH, USA; ^3^ Comprehensive Cancer Center, The Ohio State University, Columbus, OH, USA; ^4^ William G. Lowrie Department of Chemical and Biomolecular Engineering, The Ohio State University, Columbus, OH, USA; ^5^ Greenebaum Cancer Center, University of Maryland, Baltimore, MD, USA; ^6^ Division of Pharmaceutics, College of Pharmacy, The Ohio State University, Columbus, OH, USA; ^7^ Gehr Family Leukemia Center, City of Hope Comprehensive Cancer Center, Duarte, CA, USA

**Keywords:** microRNA, nanoparticles, RAS, miR-181a, acute myeloid leukemia

## Abstract

Deregulation of microRNAs' expression frequently occurs in acute myeloid leukemia (AML). Lower *miR-181a* expression is associated with worse outcomes, but the exact mechanisms by which *miR-181a* mediates this effect remain elusive. Aberrant activation of the RAS pathway contributes to myeloid leukemogenesis. Here, we report that *miR-181a* directly binds to 3′-untranslated regions (UTRs); downregulates KRAS, NRAS and MAPK1; and decreases AML growth. The delivery of *miR-181a* mimics to target AML cells using transferrin-targeting lipopolyplex nanoparticles (NP) increased mature *miR-181a*; downregulated KRAS, NRAS and MAPK1; and resulted in decreased phosphorylation of the downstream RAS effectors. NP-mediated upregulation of *miR-181a* led to reduced proliferation, impaired colony formation and increased sensitivity to chemotherapy. Ectopic expression of KRAS, NRAS and MAPK1 attenuated the anti-leukemic activity of *miR-181a* mimics, thereby validating the relevance of the deregulated *miR-181a*-RAS network in AML. Finally, treatment with *miR-181a*-NP in a murine AML model resulted in longer survival compared to mice treated with scramble-NP control. These data support that targeting the RAS-MAPK-pathway by *miR-181a* mimics represents a novel promising therapeutic approach for AML and possibly for other RAS-driven cancers.

## INTRODUCTION

Acute myeloid leukemia (AML) is a complex neoplastic disease of the hematopoietic system resulting in maturation arrest and aberrant proliferation of leukemic cells. Despite the use of cytogenetic and molecular risk stratification for treatment guidance, the majority of AML patients still do not achieve long-term survival. A better knowledge of the disease biology and novel targeted therapeutic approaches may improve cure rates.

Recently, we and others reported that the deregulated expression of microRNAs (miRs) – small non-coding RNA molecules regulating post-transcription protein expression – is associated with AML [1, 2]. Assessing the expression levels of some miRs refines patients' molecular risk classification and helps selecting treatment regimens [1–11]. These results are being translated into the clinic, and early clinical trials targeting miRs have been initiated [12–16].

The *miR-181* family comprises four mature miRs (*miR-181a*, *miR-181b*, *miR-181c*, *miR-181d*) and has been associated with the regulation of inflammatory mechanisms [17, 18]. Physiologically, *miR-181* may accelerate the megakaryocyte differentiation of CD34-positive hematopoietic cells [19]. Furthermore, these miRs have been found to be deregulated in several types of human cancers, including leukemias [2, 9, 20–26]. In solid tumors the role of *miR-181* seems to be organ-specific. High expression of *miR-181* has been associated with poor clinical outcomes in patients with colorectal cancer [20] and lymph node metastasis in oral squamous cell carcinoma [21]. However, in glioma high expression of *miR-181* seems to have tumor suppressor activity [22]. In hematologic malignancies higher expression of *miR-181* is associated with better outcomes [2, 9, 26–28]. Indeed, we recently reported the favorable impact of higher *miR-181a* expression in both AML cytogenetically normal (CN) or abnormal (CA) patients [2, 9, 28]. To date, however the molecular basis for the attenuation of disease aggressiveness by *miR-181a* remains to be fully elucidated.

RAS proto-oncogenes encode small GTPase proteins, that is, KRAS, NRAS and HRAS, that are involved in homeostatic mechanisms of proliferation, differentiation and apoptosis of normal cells [29]. Whereas *KRAS* and *NRAS* are frequently mutated and activated in AML, *HRAS* mutations are rare, and *HRAS* wild-type expression is the lowest with respect to the other RAS isoforms in the hematopoietic system [29]. Aberrant activation of RAS signal transduction is often found in human neoplasia [30–43]. In hematopoietic malignancies, including AML, activating oncogenic RAS mutations contribute to malignant phenotypes by phosphorylating and activating downstream effectors such as the mitogen-activated protein kinase kinase (MAPKK, also known as MEK), mitogen-activated protein kinase (MAPK), and the PI3K-AKT downstream effectors, thereby promoting aberrant cell proliferation and survival [29]. However, to date, an effective therapeutic approach targeting RAS directly remains to be developed.

Recently, *KRAS* was shown to be a direct *miR-181a* target in oral squamous cell carcinoma [44]. Additionally, *NRAS* and the RAS-downstream effector *MAPK1* are *in silico* predicted to be putative *miR-181a* targets. We hypothesized that higher *miR-181a* levels attenuate AML aggressiveness by targeting RAS and/or its downstream effectors in myeloid blasts, thereby reducing proliferation and decreasing the apoptotic threshold. Therefore, we reasoned that the delivery of synthetic *miR-181a* mimics may increase the low endogenous levels of *miR-181a* in AML blasts and lead to anti-leukemic activity.

## RESULTS

### Anti-leukemic activity of *miR-181a*

We previously reported that chemotherapy-treated patients with AML with higher *miR-181a* expression achieved complete remission (CR) more frequently and had longer survival compared to lower *miR-181a* expressing patients [2, 9]. In line with these clinical observations, we and others showed that *miR-181a* expression is associated with a higher sensitivity to cytarabine in AML cell lines [45, 46].

**Figure 1:**
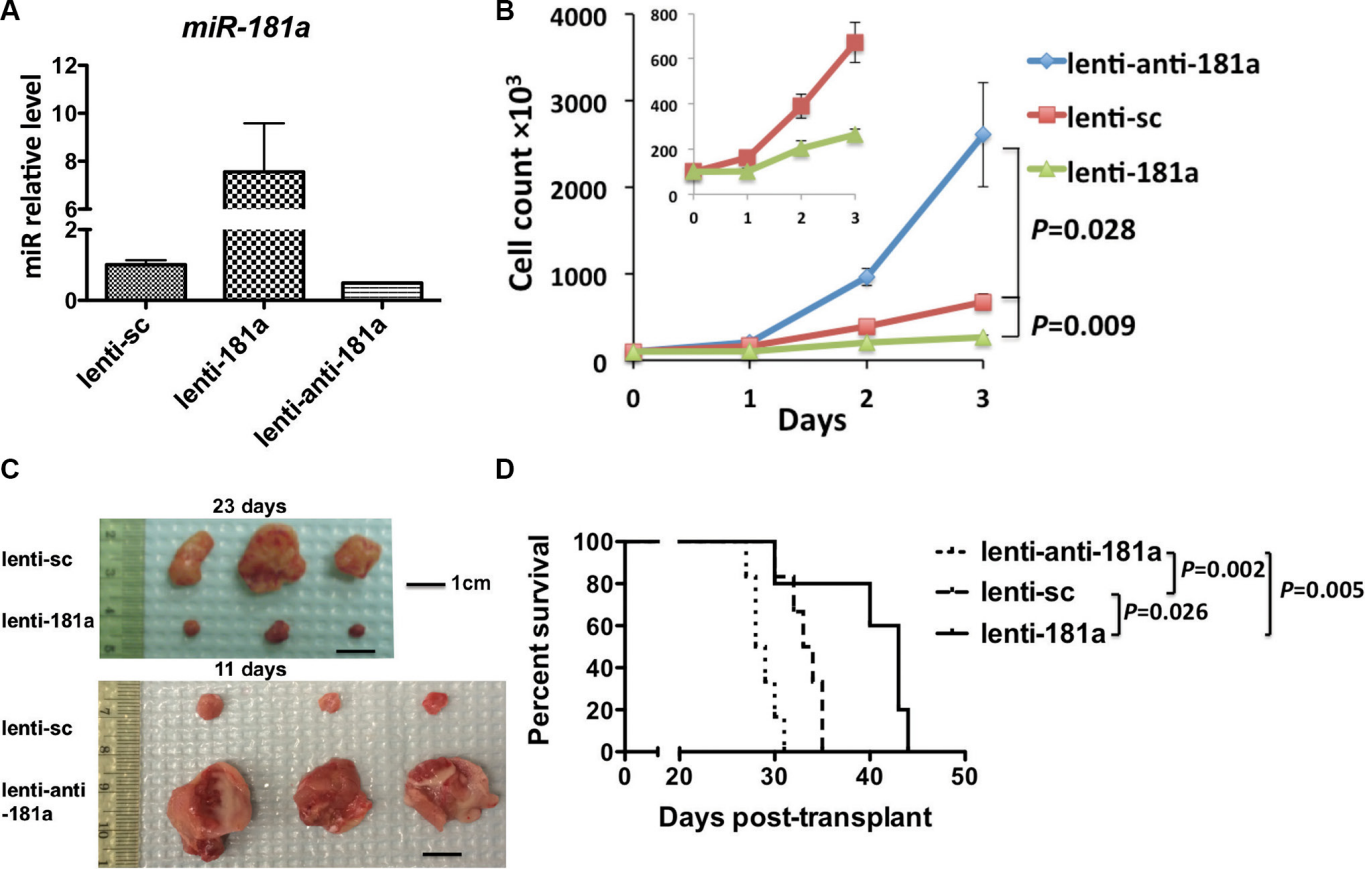
Higher levels of *miR-181a* are associated with a less aggressive phenotype in AML cells and longer survival in a murine AML model (**A**) *miR-181a* expression in MV4-11 cells after lentiviral infection. (**B**) Growth curve of MV4-11 cells transduced with lentiviral constructs either overexpressing *miR-181a* (lenti-181a), expressing a scramble sequence (lenti-sc; = control) or a knock-down construct of *miR-181a* (lenti-anti-181a). (**C**) Five million lentiviral transduced cells were engrafted subcutaneously in NOD/SCID mice. At day 11, tumors from lenti-anti-181a and lenti-sc group (*n* = 3 in each group) were isolated and weighed (no tumor in lenti-181a group). At day 23, tumors from lenti-181a and lenti-sc group (*n* = 3 in each group) were isolated and weighed. (**D**) 1.5 million lentiviral transduced MV4-11 cells were engrafted into NSG mice. Survival curves of the mice in the three groups.

These findings led us to postulate a tumor suppressor activity of *miR-181a* that we first tested by overexpressing or knocking-down *miR-181a* in the FLT3-ITD positive MV4-11 AML cell line by lentiviral infection (Figure 1A). Overexpression of *miR-181a* (lenti-*181a*) inhibited cell growth (Figure 1B; lenti-*181a* vs. lenti-sc: *P* = 0.009), whereas downregulation of *miR-181a* (lenti-*anti-181a*) enhanced cell proliferation compared to cells transfected with a vector carrying a scramble sequence (lenti-sc) (Figure 1B; lenti-sc vs. lenti-*anti-181a*: *P* = 0.028). We next engrafted 5 × 10^6^ virally transduced MV4-11 cells into NOD/SCID mice subcutaneously (*n* = 3 in each group). On day 11, the average tumor weights for animals engrafted with the lenti-*anti-181a* or lenti-sc transduced cells were 1.642 ± 0.65 g and 0.076 ± 0.022 g, respectively (Figure 1C). No tumor growth was evident in animals engrafted with lenti-*181a* transduced cells. On day 23, the average tumor weights for the lenti-sc and the lenti-*181a* transduced cell-engrafted groups were 0.65 ± 0.49 g and 0.037 ± 0.025 g, respectively (Figure 1C).

To further support the putative tumor suppressor activity of *miR-181a*, we engrafted NSG mice with virally transduced MV4-11 cells through a tail vein. The median survival for the animals engrafted with the lenti-*miR-181a*, lenti-sc and lenti-*anti-181a* transduced cells were 43, 33.5 and 28.5 days, respectively (Figure 1D). Compared to the control group (lenti-sc), the lenti-*anti-181a* mice lived significantly shorter (*P* = 0.002, log-rank test) and lenti-*miR-181a* mice significantly longer (*P* = 0.02). Though the mice in three groups showed survival time differences, they all died from AML-like disease (Supplementary Figure S1).

We concluded that higher *miR-181a* expression leads to a less aggressive AML phenotype, thereby functionally validating the previously reported prognostic results [2, 9, 28].

### KRAS, NRAS and MAPK1 are direct targets of *miR-181a*

**Figure 2:**
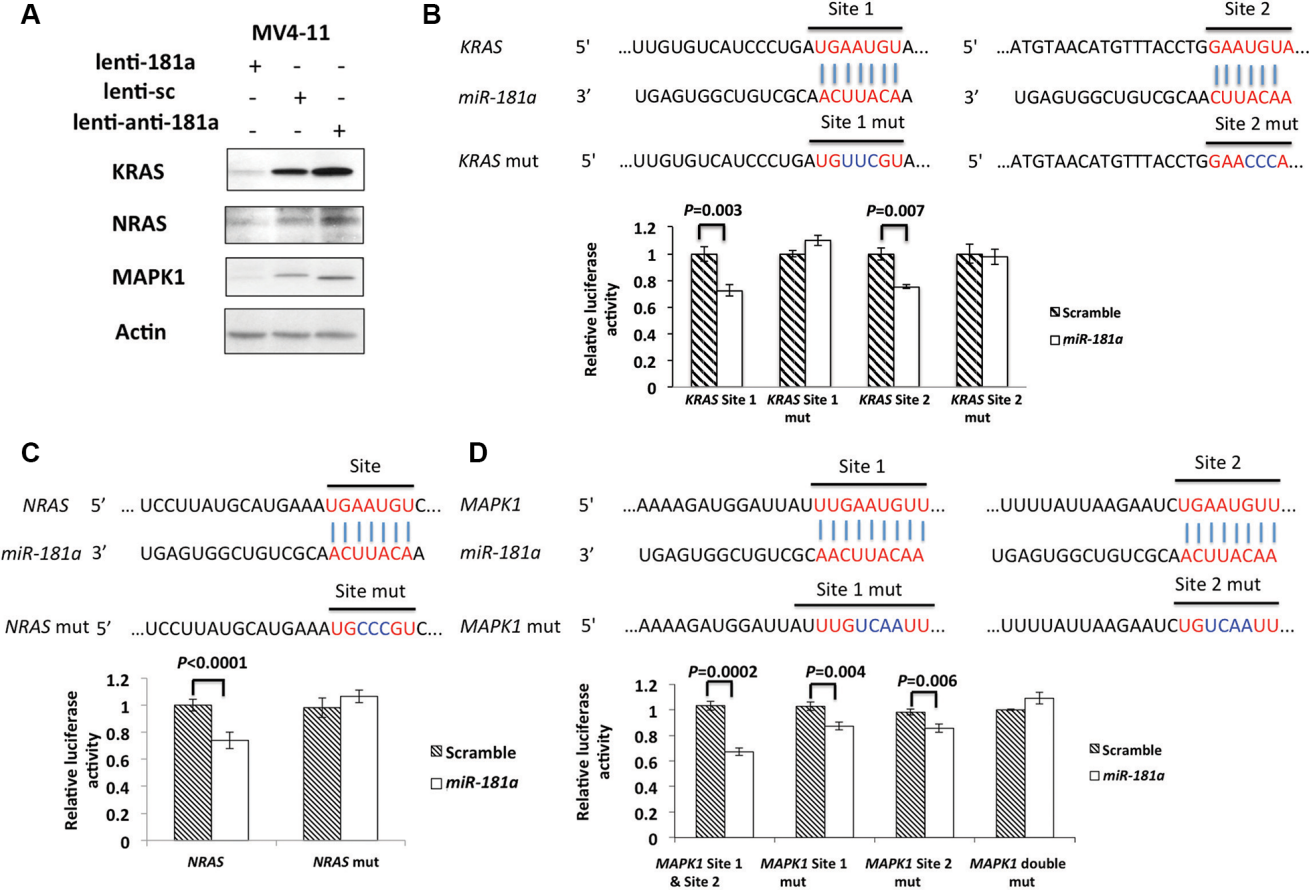
NRAS, KRAS and MAPK1 are direct targets of *miR-181a* (**A**) KRAS, NRAS and MAPK1 protein expression in infected MV4-11 and OCI-AML3 cells with lenti-181a, lenti-sc or lenti-anti-181a. Dual luciferase assays of HEK293T cells co-transfected with firefly luciferase constructs containing the *KRAS* (**B**), *NRAS* (**C**) and *MAPK1* (**D**) wild-type or mutated 3′-UTRs and *miR-181a* mimics or scramble mimics (as controls). The firefly luciferase activity was normalized to Renilla luciferase activity. The data are shown as relative luciferase activity of *miR-181a* mimic transfected cells with respect to the scramble control of nine data points from three independent transfections. Error bars represent the standard deviation (SD).

The RAS-MAPK1 and RAS-AKT-pathways are often aberrantly activated in AML and are known to contribute to myeloid leukemogenesis [29–43]. *KRAS* has been shown to be a direct *miR-181a* target in oral squamous cell carcinoma [44]. Here, we first tested whether *KRAS* and other genes involved in these pathways, including *NRAS* and its downstream effectors (i.e., *MAPK1*), were *miR-181a* targets in AML. Utilizing *in silico* tools (targetscan.org, http://diana.imis.athena-innovation.gr/ and microrna.org) we first identified putative *miR-181a*-binding sites in the 3′-untranslated regions (3′-UTRs) of *KRAS*, *NRAS* and *MAPK1*. In contrast, we could not identify putative *miR-181a* binding sites in the 3′-UTR of *HRAS*, which is rarely mutated in AML. We then tested whether *miR-181a* was able to reduce the expression of these genes in AML cells. *miR181a* overexpression by a lenti-*181a* vector reduced KRAS, NRAS, and MAPK1 protein levels 5.2, 2.1, and 6.5-fold, respectively, compared to scramble expressing controls in MV4-11 cells (Figure 2A). Consistent with these results, knock-down of *miR-181a* by a lenti-*anti-181a* increased KRAS, NRAS and MAPK1 1.5, 1.5 and 1.8-fold compared to scramble controls (Figure 2A).

Next we showed that the modulation of KRAS, NRAS and MAPK1 expression by *miR-181a* was caused by direct binding to the respective 3′-UTRs. We first validated *KRAS* as a direct *miR-181a* target. We identified two *miR-181a*-binding sites in the *KRAS* 3′-UTR and observed a 28 ± 4% (*P* = 0.003) and a 25 ± 1% (*P* = 0.007) downregulation of luciferase activity on site 1 and site 2 after co-transfecting 293T cells with *miR-181a* compared with scramble expressing controls. Mutations in the seed sequences of the *KRAS* 3′-UTRs rescued the *miR-181a*-induced downregulation (Figure 2B). Next, to demonstrate that *NRAS* is also a direct *miR-181a* target, we cloned the predicted *miR-181a*-binding-site in the *NRAS* 3′-UTR into a luciferase reporter, and we observed a 26 ± 6% (*P* < 0.0001) downregulation of luciferase activity. An introduced mutation in the seed sequence rescued the *miR-181a*-induced downregulation (Figure 2C). We also identified two putative *miR-181a* binding sites in the *MAPK1* 3′-UTR. Because of the short distance between the two binding sites (149 base pairs), we cloned the two binding sites into the same luciferase reporter construct. We observed a 33 ± 2% (*P* = 0.0002) downregulation of luciferase activity with *miR-181a* treatment compared to cells with scramble control treatment. When we mutated the two sites separately, we observed a 13 ± 3% (site 1; *P* = 0.004) and a 15 ± 3% (site 2; *P* = 0.006) *miR-181a*- induced downregulation of the luciferase activity. However, mutations on both sites of *MAPK1* could completely rescue the *miR-181a*-induced downregulation (Figure 2D). Collectively, these results support that KRAS, NRAS and MAPK1 are direct *miR-181a* targets.

### Delivery of synthetic *miR-181a* mimic by transferrin (Tf)-conjugated nanoparticles (NP) enhanced *miR-181a* levels and inhibited RAS-dependent signaling pathways in AML

**Figure 3:**
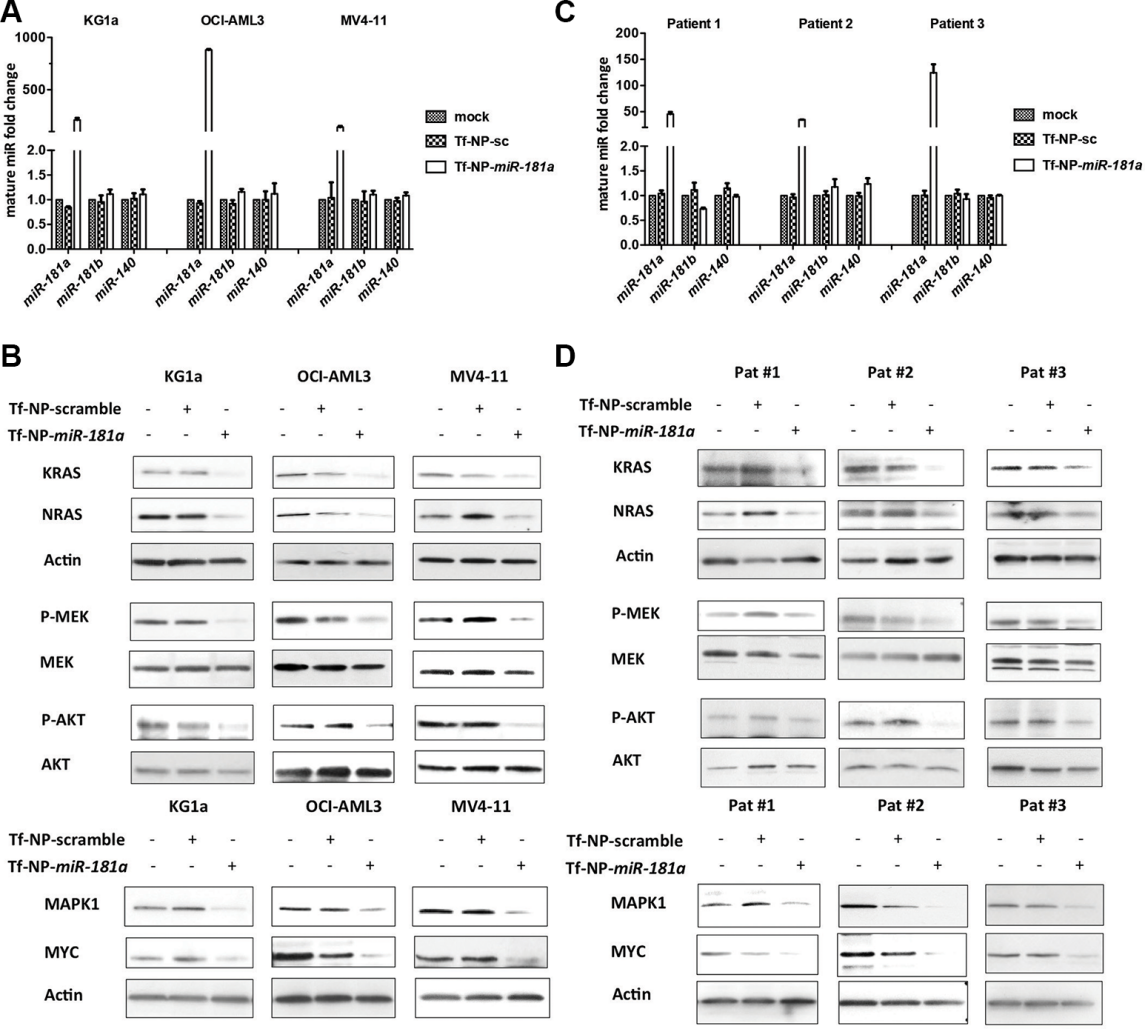
Treatment with Tf-NP-*miR-181a* increased mature *miR-181a* levels; downregulated KRAS, NRAS, and MAPK1; and inhibited the RAS-MAPK1 signaling pathway Mature *miR-181a*, *miR-181b* and *miR-140* expression levels in KG1a, OCI-AML3 and MV4-11 cells (**A**) and primary patient blasts (*n* = 3) (**C**). NRAS, KRAS, p-MEK, MEK, p-AKT, AKT, MAPK1, and MYC expression in KG1a, OCI-AML3 and MV4-11 cells (**B**) and primary patient blasts (*n* = 3) (**D**) treated with mock, Tf-NP-sc and Tf-NP-*miR-181a*.

Because higher *miR-181a* levels are associated with improved outcomes in AML [2, 9, 26–28], and because *miR-181a* downregulation contributed to leukemia growth (Figure 1) and directly targeted KRAS, NRAS and MAPK1, we reasoned that increasing *miR-181a* may have therapeutic value in AML. We have previously demonstrated the successful delivery of miR mimics to AML blasts via transferrin (Tf)-targeted anionic lipid-based lipopolyplex nanoparticles (NP) [47]. Here, we used a similar approach to deliver synthetic *miR-181a* mimics. We chose KG1a, MV4-11 and OCI-AML cells as models because of the relatively low *miR-181a* levels and activated RAS pathways (Supplementary Figure S2). Following treatment with Tf-NPs encapsulating *miR-181a* double-stranded mimic molecules (Tf-NP-*miR-181a*; 10 nM) or scramble control molecules (Tf-NP-sc; 10 nM), levels of mature *miR-181a* were measured by qRT-PCR. After 24 hours exposure, mature *miR-181a* levels increased 211 ± 31, 880 ± 10 and 142 ± 10-fold in KG1a, OCI-AML3 and MV4-11 cells, respectively, whereas levels of *miR-181b* and unrelated *miR-140* remained unchanged (Figure 3A).

Having shown that the Tf-NP-delivery-system was able to deliver *miR-181a* to AML blasts, we next tested the impact of Tf-NP-*miR-181a* on RAS activity. First, we found that the delivered synthetic *miR-181a* was functional, as it downregulated KRAS, NRAS and MAPK1 proteins (KG1a: 4.3, 4.4 and 5.5-fold; OCI-AML3: 3.2, 3.9 and 2.2-fold; MV4-11: 1.5, 4.4 and 4.6-fold, respectively) compared to Tf-NP-sc treatment (Figure 3B). Compared to Tf-NP-sc, Tf-NP-*miR-181a* decreased p-MEK protein by 6.8, 2.2 and 4.5-fold and p-AKT protein by 2.0, 2.5 and 5.7-fold in KG1a, OCI-AML3 and MV4-11 cells, respectively (Figure 3B). Finally, we assessed the expression of the oncogenic transcription factor MYC, whose protein stability is enhanced by the RAS-MAPK1 phosphorylation pathway [48]. There was a 4.8, 4.3 and 7.8-fold reduction of MYC protein in KG1a, OCI-AML3 and MV4-11 treated with Tf-NP-*miR-181a* compared to those treated with Tf-NP-sc control (Figure 3B). Consistent with these results, anti-*miR-181a* treatment resulted in upregulation of the KRAS, NRAS and MAPK1 proteins in HL60 cells that present with higher levels of endogenous *miR-181a* (Supplementary Figures S2A and S3).

To validate these results, we treated primary AML blasts having activated RAS from three AML patients (Patient No 1-3; Supplementary Table S2) (Supplementary Figure S2) with Tf-NP-*miR-181a* and again observed an increase in *miR-181a* (Figure 3C). After 24 hours, mature *miR-181a* levels increased 45 ± 4, 35 ± 0.1 and 125 ± 16-fold, respectively, in the three patient blasts samples treated with Tf-NP-*miR-181a* compared to the Tf-NP-sc treated controls, whereas levels of *miR-181b* and *miR-140* remained unchanged (Figure 3C). Increased levels of *miR-181a* resulted in decreased protein levels of KRAS, NRAS and MAPK1 by 6.3, 6.8 and 5.6-fold in patient 1; 6.4, 1.6 and 19.7-fold in patient 2; and 2.3, 2.4 and 3.4-fold in patient 3, respectively (Figure 3D). Downregulation of RAS and MAPK1 resulted in RAS-MAPK1 inhibition, decreased MEK and AKT phosphorylation and decreased MYC levels. We observed a 1.4, 3.5 and 2.0-fold decrease of p-MEK, 1.8, 9.3 and 2.0-fold decrease of p-AKT, as well as a 5.3, 7.6 and 2.8-fold decrease of MYC normalized in the patient blasts treated with Tf-NP-*miR-181a* compared to Tf-NP-sc treatment (Figure 3D).

In summary, we showed the effective delivery of *miR-181a* via Tf-conjugated nanoparticles and in turn downregulation of KRAS, NRAS and MAPK1 and inhibition of the RAS-MAPK1 and RAS-AKT-kinase signaling cascade.

### Tf-NP-*miR-181a* treatment in AML cells

**Figure 4:**
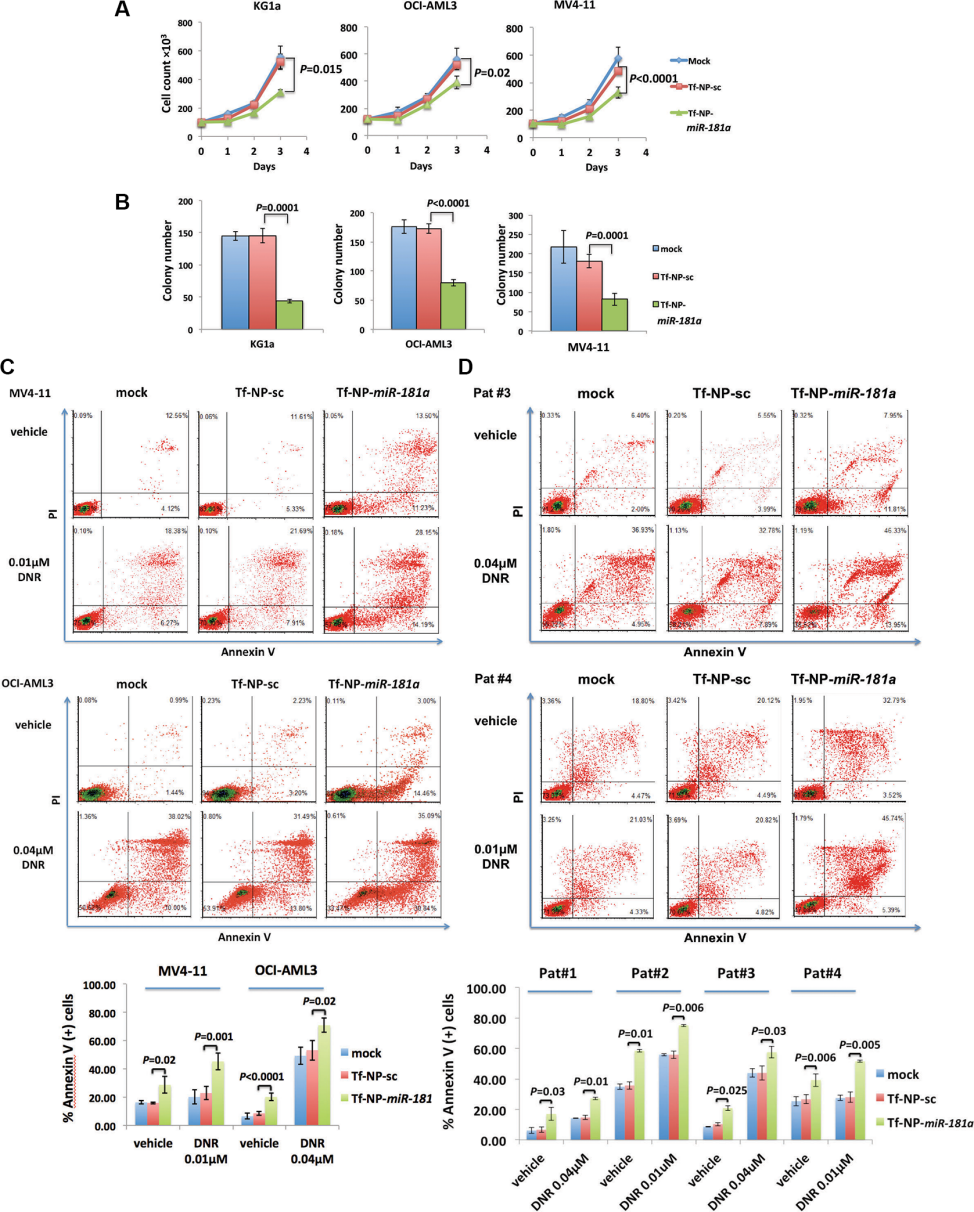
Treatment with Tf-NP-*miR-181a* had anti-leukemic activity in AML cells Cell growth curve (**A**) and colony numbers (**B**) of KG1a, OCI-AML3 and MV4-11 cells treated with Tf-NP-*miR-181a*, Tf-NP-sc or mock. Error bars represent SD. Annexin V assays in MV4-11 and OCI-AML3 cells (**C**) as well as patient blast cells (**D**) treated with Tf-NP-*miR-181a*, Tf-NP-sc or mock in the presence or absence of daunorubicin (DNR, 0.01 μM for MV4-11, 0.04 μM for OCI-AML3, 0.01 μM for patient 1 [Pat #1] and patient 3 [Pat #3], 0.04 μM for patient 2 [Pat #2] and patient 4 [Pat #4] blasts). DNR was added 24 hours after priming cells with nanoparticle-miR treatment for another 72 hours.

Next, we demonstrated the anti-leukemic activity of the Tf-NP-*miR-181a*, which led to reduced proliferation of KG1a cells by 40% (*P* = 0.015), OCI-AML3 cells by 25% (*P* = 0.023) and MV4-11 cells by 32% (*P* < 0.0001) after 72 hours compared to Tf-NP-sc control (Figure 4A). To validate the RAS-MAPK1 and RAS-AKT-kinase-pathways as relevant anti-leukemic *miR-181a* targets, we treated KG1a and MV4-11 cells with Tf-NP loaded with siRNAs for *KRAS*, *NRAS* and *MAPK1* (Supplementary Figure S4A). Following this treatment, we observed a similar anti-leukemic effect. The combined siRNA treatment reduced proliferation of KG1a cells by 32% and MV4-11 cells by 30% compared with scramble siRNA treatment (Supplementary Figure S4B). The reduced proliferation induced by Tf-NP-*miR-181a* treatment was reversed by lentiviral expression of *KRAS*, *NRAS* and *MAPK1* in OCI-AML3 cells (Supplementary Figure S5A–S5C; Supplementary Table S3) attenuating the anti-leukemic activity of Tf-NP-*miR-181a* and thereby supporting the relevance of these targets to leukemogenesis. We also observed a more than 50% reduction of colony formation following Tf-NP-*miR-181a* treatment after 2 weeks (Figure 4B). The average number of colonies formed with mock treatment (buffer only), Tf-NP-sc control and Tf-NP-*miR-181a* treatment were, respectively, 145 ± 7, 145 ± 11 and 44 ± 3 (*P* = 0.0002 compared to Tf-NP-sc) for KG1a, 176 ± 11, 172 ± 8 and 80 ± 6 (*P* < 0.0001 compared to Tf-NP-sc) for OCI-AML3 and 217 ± 42, 180 ± 17 and 82 ± 15 (*P* = 0.0001 compared to Tf-NP-sc) for MV4-11.

Treatment with Tf-NP-*miR-181a* induced apoptosis in both MV4-11 (28.69 ± 5.88% vs. 15.92 ± 0.7% annexinV+, *P* = 0.02) and OCI-AML3 cells (20.15 ± 2.58% vs. 8.54 ± 1.42% annexinV+, *P* < 0.0001) compared to Tf-NP-sc treatment at 96 hours (Figure 4C). Following a combined siRNA treatment with Tf-NP loaded with siRNAs for *KRAS*, *NRAS* and *MAPK1*, we observed similar effects in MV4-11 and OCI-AML3 cells (Supplementary Figure S4C). In addition, after 24 hours of priming cells with *miR-181a*, daunorubicin (DNR) was added to treat the cells for another 72 hours. We observed that *miR-181a* treatment enhanced the apoptotic effect of DNR in MV4-11 (*miR-181a* - > 0.01 μM DNR: 45.27 ± 5.99% vs. scramble - > 0.01 μM DNR: 22.88 ± 4.61% annexinV+, *P* = 0.001) and OCI-AML3 (*miR-181a* - > 0.04 μM DNR: 70.92 ± 5.01% vs. scramble - > 0.04 μM DNR: 53.25 ± 7.06% annexinV+, *P* = 0.02; Figure 4C). We also observed similar effects priming MV4-11 and OCI-AML3 cells with siRNAs for *KRAS*, *NRAS* and *MAPK1* (Supplementary Figure S4C). We then validated our observation in primary patient blasts. Tf-NP-*miR-181a* induced apoptosis in all four patient blast samples compared to Tf-NP-sc controls (patient 1: 17.04 ± 4.22% vs. 6.66 ± 1.73% annexinV+, *P* = 0.03; patient 2: 58.53 ± 0.81% vs. 35.73 ± 2.41% annexinV+, *P* = 0.01; patient 3: 20.86 ± 1.55% vs. 10.32 ± 1.1% annexinV+, *P* = 0.025; patient 4: 39.28 ± 4.19% vs. 26.70 ± 2.95% annexinV+, *P* = 0.006; Figure 4D). When exposed to DNR for 72 hours, the Tf-NP-*miR-181a* treated cells exhibited increased apoptosis compared with control cells (patient 1 exposed to 0.04 μM DNR: 27.28 ± 0.87% vs. 14.75 ± 1.36% annexinV+, *P* = 0.01; patient 2 exposed to 0.01 μM DNR: 75.16 ± 0.71 vs. 55.91 ± 2.42% annexinV+, *P* = 0.006; patient 3 exposed to 0.04 μM DNR: 57.61 ± 3.77% vs. 43.99 ± 4.7% annexinV+, *P* = 0.03; patient 4 exposed to 0.01 μM DNR: 51.61 ± 0.68% vs. 28.06 ± 3.42% annexinV+, *P* = 0.005; Figure 4D).

### Systemic delivery of Tf-NP-miR-181a in an AML mouse model

**Figure 5:**
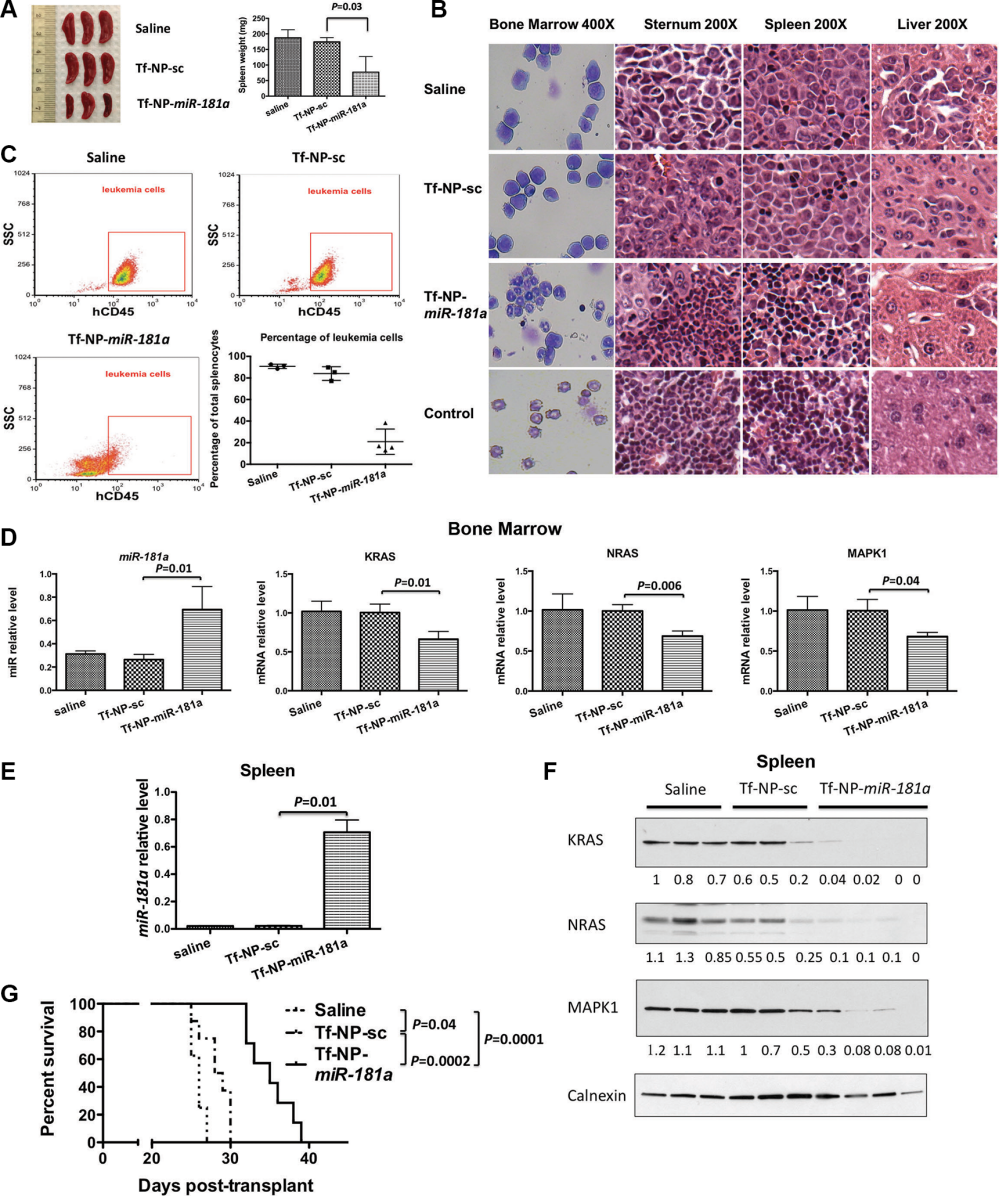
*In vivo* evaluation of Tf-NP-*miR-181a* treatment (**A**) Spleens and spleen weights from mice sacrificed after 8 doses of treatment from each group: saline, Tf-NP-sc and Tf-NP-*miR-181a* (*n* = 3). (**B**) May-Grünwald/Giemsa staining of bone marrow cells and H&E staining of sections from sternum, spleen and liver of MV4-11 engrafted mice treated with saline, Tf-NP-sc and Tf-NP-*miR-181a*. NSG mice without MV4-11 engraftment were also used as controls. (**C**) Leukemic cell population from the spleens harvested from differently treated mice and assessed by flow cytometry. (**D**) Mature *miR-181a* levels and *KRAS*, *NRAS* and *MAPK1* RNA levels in sorted MV4-11 cells from bone marrow samples harvested from differently treated mice. Error bars represent SD. (**E**) Mature *miR-181a* levels in sorted MV4-11 cells from spleens harvested from differently treated mice. Error bars represent SD. (**F**) KRAS, NRAS and MAPK1 protein expression in sorted MV4-11 cells from spleens harvested from differently treated mice. (**G**) Survival curves of the mice according to the indicated treatment.

Next, we examined the anti-leukemic activity of Tf-NP-*miR-181a in vivo*. Saline (control), Tf-NP-sc or Tf-NP-*miR-181a* were administrated (1.5 mg/kg/d miR three times/week) through a tail vein 10 days after the engraftment of MV4-11 cells in NSG mice (each group *n* = 11). Randomly, three mice from each group (i.e. saline, Tf-NP-sc or Tf-NP-*miR-181a* treated group) were sacrificed after eight treatment doses. The spleen weights were measured and resulted in 187.3 ± 25.93 mg, 174.3 ± 13.65 mg and 77 ± 50 mg (vs. Tf-NP-sc; *P* = 0.03) in the saline, Tf-NP-sc and Tf-NP-*miR-181a* groups, respectively (Figure 5A). The spleen weight was 58.3 ± 10.5 mg for age-matched blank control mice (Supplementary Figure S6). Cytospins of bone marrow cells and histopathology of sternum, spleen and liver sections from MV4-11 cell engrafted mice treated with either saline or Tf-NP-sc showed infiltration of blast cells. In contrast, cytospins of bone marrow cells and histopathology of sternum, spleen and liver from Tf-NP-*miR-181a* treated leukemic mice were similar to that of the age-matched blank control groups (Figure 5B). Furthermore, the population of leukemic cells in spleen samples, measured by flow cytometry, was significantly reduced in mice treated with Tf-NP-*miR-181a* compared to mice treated with Tf-NP-sc or saline (Figure 5C). We observed a 2.6-fold and a 35-fold increase of *miR-181a* levels in MV4-11 cells harvested and sorted from bone marrow and spleens, respectively, in the Tf-NP-*miR-181a* treated mice compared to Tf-NP-sc (Figure 5D and 5E). In these cells, RAS and MAPK1 proteins were downregulated in the Tf-NP-*miR-181a* treated mice (Figure 5D and 5F).

The median survival time of the remaining mice was 26, 28.5 and 35 days for the animal groups treated with saline, Tf-NP-sc and Tf-NP-*miR-181a*, respectively. Tf-NP-*miR-181a* treatment significantly reduced the disease burden and prolonged survival compared to Tf-NP-sc (*P* = 0.0002) or saline (*P* = 0.0001) treatment (Figure 5G). Interestingly, Tf-NP-sc treatment also had some minor anti-leukemic effects compared to the saline treated control group (*P* = 0.04).

## DISCUSSION

MiRs have been implicated in leukemogenesis, and the expression levels of several miRs have been shown to impact the prognosis of AML patients [1–9, 12–14]. Relatively low expression of *miR-181a* is associated with worse outcomes in AML patients [2, 9, 28]. Here, we provided evidence that AML cells with reduced levels of *miR-181a* had a more aggressive AML phenotype, and we validated this clinical observation functionally.

In other types of cancers *miR-181a* has been associated with both tumor suppressor and oncogene functions [20–28], implying context-specific effects. Whereas in colorectal cancer [20] and lymph node metastasis in oral squamous cell carcinoma [21] a high *miR-181* level seems to be associated with worse clinical outcomes, in glioma this miR has tumor suppressor function [22]. In these brain tumors *miR-181a* was shown to target the anti-apoptotic genes *BCL2* and *MCL1*, and downregulated *miR-181a* reduced glucose deprivation-induced apoptosis and caused mitochondrial dysfunction in astrocytes [22, 49, 50]. The *miR-181*-family has been reported to be an effector in inflammatory response by TNF-α, IL-6, IL-1β, IL-8 and IL-10 [17, 18, 51–53]. With regard to AML, we previously provided preliminary evidence that *miR-181* may target elements of the “inflammasome” that ultimately lead to NF-κB activation and leukemia growth, while Li *et al.* showed that *miR-181* promoted apoptosis, reduced viability and delayed leukemogenesis in MLL-rearranged AML by downregulating the homeobox gene *PBX3* [28]. Bai *et al.* also demonstrated that *miR-181a* may reduce BCL2 and thus enhance chemosensitivity of AML cells [46]. However, the mechanisms through which *miR-181a* attenuates disease aggressiveness and the full spectrum of its targets still remain to be fully understood in AML.

Here, we first demonstrated that *miR-181a* targets the RAS-MAPK1 and RAS-AKT pathways, which have been found to be activated and support AML leukemogenesis [54–58]. Despite extensive efforts, the direct therapeutic targeting of these pathways with small molecule inhibitors remains challenging [59]. Our results show that KRAS, NRAS and MAPK1 proteins may be effectively reduced by utilizing RNA compounds mimicking *miR-181a*. The efficient delivery of *miR-181a* mimics by Tf-NPs decreased the targets and their downstream effectors (AKT, MEK, MYC). Altogether, our results support *miR-181a* replacement as a potential anti-leukemic, RAS targeting strategy in AML. The therapeutic advantage of using miR mimics is in the simultaneous targeting of cross-talking signal transduction pathways (STPs) [38]. Although a use of synthetic mimics may be of relatively difficult in therapeutic application especially compared to the use of anti-miR oligonucleotide, it has been postulated for several types of cancers and is currently being tested in clinical trials (e.g. for *miR-34* in NCT01829971). One of the limitations of miR-based therapies is in the optimal delivery of these oligonucleotides as they are subject to rapid hepatic uptake and metabolism and are easily degraded by endonucleases in biological matrices. Nevertheless, we recently reported a novel anionic lipopolyplex nanocarrier system that was designed for the purpose of allowing for efficient miR delivery to AML cells [47]. Here we show that this system could be adapted to the delivery of *miR-181a* mimics and exert an efficient inhibitory effect on the RAS-MAPK1 and RAS-AKT kinase pathways, thereby resulting in a significant anti-leukemic activity. Interestingly, a very mild anti-leukemic effect and a slight downregulating effect of Tf-NP-scramble treatment on NRAS, KRAS, and MAPK1, as well as on MEK phosphorylation and MYC expression in OCI-AML3 cells was observed. This effect was likely mediated by one of the components of our nanoparticle system, for example, linoleic acid. It has been reported that some fatty acids have anti-tumor activity [60–62].

Other strategies to increase *miR-181a* have also been tested by our group with significant results. In a previous study, we demonstrated that lenalidomide increases endogenous *miR-181a* [45], by enhancing the expression of C/EBPα isoforms, which bind to the *miR-181a* promoter and induce the transcription of *miR-181a*. However, lenalidomide has several unwanted side-effects at the doses necessary to achieve plasma concentrations at which *miR-181a* was increased. Thus, the targeting NPs that we reported here may present the advantage to be more specifically directed to AML blasts, thereby sparing normal tissues and perhaps reducing unwanted toxicity. Our preclinical studies showed encouraging results with no toxicity in NP-treated mice at doses inducing anti-leukemic effects [47]. It should also be underscored that we and others have reported that increased levels of miR-181 lead to enhancement of sensitivity to chemotherapy in AML models [45, 46, 63]. Furthermore, patients with higher levels of miR-181a have a better complete remission rate and longer survival compared with those with lower levels, further supporting a role of this miR as a modifier of the response to chemotherapy [9]. Thus, we envision that potential clinical benefit of *miR-181a* replacement will be more likely if applied in combination with chemotherapy.

In summary, we unveil here a previously unreported activity of *miR-181a* that directly downregulates NRAS, KRAS and MAPK1 and RAS-dependent downstream signals supporting leukemogenesis. We showed that a nanoparticle-based delivery system could be used to efficiently increase otherwise low levels of *miR-181a* and achieve anti-leukemic activity in AML models with no evident toxicity. On the basis of our results, *miR-181a*-NP may warrant further evaluation for potential clinical applications in AML and other RAS-dependent malignancies.

## MATERIALS AND METHODS

### Cell lines and patient samples

KG1a, MV4-11, HL60, HEK 293T and HEK 293TN cells were obtained from ATCC (Manassas, VA); OCI-AML3 cells were obtained from DSMZ (Braunschweig, Germany). Primary, unselected AML blasts from apheresis samples collected from nine patients were obtained from The Ohio State University (OSU) Leukemia Tissue Bank. Patients signed an informed consent to store and use their tissue for discovery studies according to OSU institutional guidelines.

### Lentiviral infections

The lentiviral infections were performed as previously described [7]. The stemloop of *miR-181a* with 200 bp flanking sequence was cloned into the HIV based lentiviral dual promoter vector (pCDH-CMV-MCS-EF1-copGFP+Puro cDNA; System Biosciences, Mountain View, CA). The miRZip anti-*miR-181a* (lenti-anti-181a) and scramble vectors were purchased from System Biosciences.

### Luciferase assays

Luciferase assays were carried out as previously described [7]. 293T cells were co-transfected with luciferase vector (pGL4.24), Renilla control vector and *miR-181a* mimic or scramble control. Luciferase activity was normalized to Renilla activity. See supplementary material for more detailed information.

### Nanoparticle preparation and treatment

The synthetic double-stranded *miR-181a*, miR-scramble (sc), and *KRAS*, *NRAS* and *MAPK1* siRNAs were purchased from Ambion. Nanoparticle preparation was performed as previously described [47, 64, 65]. Briefly, polyethylenimine was used to capture miRs/siRNAs, and the complex was loaded to pre-made anionic liposomal nanoparticles which consists of 1,2-dioleoyl-sn-glycero-3-phosphoethanolamine (DOPE), 1,2-dimyristoyl-sn-glycerol, methoxypolyethylene glycol (DMG-PEG) and linoleic acid. Transferrin was first conjugated with 1,2-distearoyl-sn-glycero-3-phosphoethanolamine-N-[maleimide(polyethylene glycol)-2000] (DSPE-PEG2000 maleimide) and then post-inserted to the miR loaded nanoparticle to form the final product. The final concentration of the miRs/siRNAs was 10 nM and was used for all *in vitro* studies. Protein was collected at 24 and 48 hours for western blot analysis.

### Quantitative RT-PCR (qRT-PCR)

Total RNA was extracted with TRIzol reagent (Invitrogen). cDNA was synthesized using Superscript III (Invitrogen) or the Taqman miR Reverse Transcription kit (Applied Biosystems, Foster City, CA) for *miR-181a, miR-181b, miR-140* and *U44*. qRT-PCR was performed with Taqman gene expression assays (Applied Biosystems) following the manufacturer's protocols. *miR-181a, miR-181b* and *miR-140* expression were normalized to *U44*. *KRAS*, *NRAS* and *MAPK1* expression were normalized to *GAPDH*. The comparative cycle threshold (C_T_) method as previously described was used for relative quantification of gene expression [47].

### Western blot analysis

Anti-KRAS (ab55391) antibodies were purchased from Abcam (Cambridge, MA). Anti-NRAS (C-20, sc-519) and Anti-MYC (N-262, sc-764) antibodies were purchased from Santa Cruz Biotechnology (Santa Cruz, CA). Anti-MAPK1, –MEK1/2 (L38C12), –p-MEK1/2 (S217/221,41G9), AKT and p-AKT (S473, D9E) antibodies were purchased from Cell Signaling Technology (Beverly, MA). Equivalent gel loading was confirmed by probing with antibodies against actin (sc-1616; Santa Cruz) or calnexin (C5C9; Cell Signaling). The intensity of the resulting bands was measured by ImageJ 1.48 s (http://imagej.nih.gov/ij). The intensity ratio of each band respective to the corresponding actin intensity was used for relative quantification.

### Growth curves

Lentivirally transduced MV4-11 cells (1 × 10^5^/mL) were plated in 12-well plates. KG1a, OCI-AML3 and MV4-11 cells (1 × 10^5^/mL) were plated in 12-well plates and treated with nanoparticles (Tf-NP-sc or Tf-NP-*miR-181a* at a final concentration of 10 nM) or were mock treated (buffer only). Cells were harvested and counted at 24-hour intervals using a Bio-Rad TC20 Automated Cell Counter (Bio-Rad, Berkeley, CA). Each sample was run in triplicate.

### Colony assays

Methylcellulose colony formation assays were carried out as previously described [66] and counted after 15 days.

### Apoptosis assays

MV4-11 and OCI-AML3 cells and four AML patient blast samples cells were treated with Tf-NP-*miR-181a,* siRNAs, Tf-NP-sc and mock for 24 hours. The cells were then subsequently treated with daunorubicin (DNR; 0.01 μM for MV4-11, 0.04 μM for OCI-AML3, 0.04 μM for patient #1 and #3, 0.01 μM for patient #2 and #4 blasts; Sigma-Aldrich, St Louis, MO) or vehicle control (phosphate-buffered saline; Sigma-Aldrich) for another 72 hours. Annexin V/propidium iodide (PI) stain (BD Biosciences, San Jose, CA) was performed.

### *In vivo* studies

Animal studies were performed according to the Ohio State University institutional guidelines. A total of 5 million lentiviral transduced MV4-11 cells were injected subcutaneously into eight-week female NOD/SCID gamma mice (NSG; The Jackson Laboratory, Bar Harbor, ME). At day 11, 3 mice from each lenti-*anti-181a* and lenti-sc group were sacrificed, and tumors were weighed. At day 23, 3 mice from each lenti-sc and lenti-*181a* group were sacrificed, and tumors were weighed.

For the functional study, six-week-old NSG mice were injected with 0.15 million lentivirally transduced MV4-11 cells intravenously through a tail vein (*n* = 6 in each group: lenti-*anti-181a*, lenti-sc and lenti-*181a*).

For the therapeutic study, six-week-old NSG mice were injected with 0.3 million MV4-11 cells intravenously through a tail vein. The treatment started 10 days after the engraftment. Mice were treated with saline, Tf-NP-sc or Tf-NP-*miR-181a* (1.5 mg/kg/d three times/week). Randomly, 3 mice of each group were sacrificed after 8 doses of treatment for pathology analysis. Age-matched NGS mice without MV4-11 cell engraftment were used as blank control. The treatment was continued for the remaining mice. Eight mice from each group were monitored for survival. The experiment was repeated for biomarker analysis. Bone marrow and spleen cells were isolated from scarified mice and sorted for human CD45-positive cells for further analysis.

### Statistical analysis

Data are presented as mean ± SD of at least 3 independent experiments and analyzed by the two-tailed Student's *t-test*. The mean and SD were calculated and displayed in bar graphs as the height and the corresponding error bar, respectively. Mouse survival was calculated using the Kaplan–Meier method, and survival curves were compared by the log-rank test.

## SUPPLEMENTARY MATERIALS TABLES AND FIGURES


