# Supplementary material for: Targeting the RAS/MAPK pathway with miR-181a in acute myeloid leukemia
Source: Oncotarget. 2016 Aug 9;7(37):59273–86. doi: 10.18632/oncotarget.11150 (PMC5312311; doi:10.18632/oncotarget.11150)
Supplement: Supplementary file 1 [file oncotarget-07-59273-s001.pdf]

# Targeting the RAS/MAPK pathway with *miR-181a* in acute myeloid leukemia

## Supplementary Materials

## SUPPLEMENTARY MATERIALS AND METHODS AND RESULTS

### Lentivirus infection

For overexpression the stemloop of *miR-181a* with 200 bp flanking sequence was cloned into the HIV based lentiviral dual promoter vector (pCDH-CMV-MCS-EF1-copGFP+Puro cDNA; System Biosciences, Montain View, CA, USA), utilizing the primers and PCR conditions described in the Supplementary Information (SI, Supplementary Table S1). For the *miR-181a* knock-down experiments the miRZip anti-miR-181a (lenti-anti-181a) was purchased from System Biosciences. Lentiviral miR scramble control was used (miRZiP000, System Biosciences). ORF clone of KRAS (NM_004985) and MAPK1 (NM_002745.4) were purchased from OriGene (Rockville, MD) and Sino Biological Inc. (Beijing, China) respectively. KRAS and MAPK1 were cloned from above mentioned plasmid and NRAS was cloned from cDNA to the HIV based lentiviral dual promoter vector. For more detailed information please see supplementary material. For each experiment 4500 µg lentiviral constructs were transfected into 293TN cells using 45 µg pPACKH1 (System Biosciences) and 55 µl PureFection (System Biosciences). After 48 hours and 72 hours, the supernatant containing the pseudoviral particles was collected and the virus was precipitated overnight at 4°C using 5 ml PEG-IT virus precipitation solution (System Biosciences). 200 µl Phosphate Buffered Saline (PBS) and 25 µM Hepes Buffer were used for resuspension of the pelleted virus. 500 000 MV4-11 cells/ml were infected with 20 UI virus, using 5 µl Transdux Infection Reagent (System Biosciences). Ten Days later the infected cells were selected with Puromycin.

### Luciferase assays

The 3′-untranslated regions (UTRs) of the *KRAS, NRAS* and *MAPK1* gene were amplified by PCR from genomic DNA and inserted into the pGL4.24 control vector (Promega, Madison, WI, USA), using the EcoRI site immediately downstream from the stop codon of the luciferase coding region. We also generated mutated binding sites by using bi-directional mutation primers, exchanging 3 or 4 nucleotides of the respective predicted binding sequences. Primer sequences are listed in Supplementary Table S1. HEK 293T cells were seeded in 12-well plates 24 hours before the transfection. When the culture reached 80% confluency, the cells were transfected in triplicates with reporter and control constructs (Renilla, pGL4.74; Promega) using the Lipofectamine 2000 transfection reagent (Invitrogen, Carlsbad, CA, USA) according to the manufacturer's instructions. Cells were co-transfected with 10 pmol of either *miR-181a* or miR-scramble control (Ambion, Austin, TX, USA). After 24 hours, protein lysates were assessed for firefly luciferase and Renilla luciferase activities according to the manufacturer's recommendations (Promega). For analysis, relative expression was normalized using co-transfected Renilla luciferase.

### *miR-181a* expression in AML cells and cell samples of healthy donors

We compared expression levels of *miR-181a* in different AML cell lines, primary blasts for AML patients and bone marrow cells from healthy donors (Supplementary Figure S2A). We found that while *miR-181a* was significantly downregulated in AML primary cells compared to bone marrow cells of healthy donors, *miR-181a* levels were comparable between AML primary patient blasts and AML cell lines.

All healthy donors of bone marrow cells signed an informed consent to store and use their tissue for discovery studies according to OSU institutional guidelines.

### Effects of *KRAS, NRAS* and *MAPK1* siRNA treatment

Following a combined siRNAs treatment with Tf-NP loaded with siRNAs for *KRAS*, *NRAS* and *MAPK1* (60 nM for each, Supplementary Figure S4A) we observed anti-proliferative (Supplementary Figure S4B)

and pro-apoptotic effects comparable to those after *miR-181a* treatment in MV4-11 (65.6 ± 5.3% vs. 15.1 ± 7.0% annexinV+, $P < 0.0001$) and OCI-AML3 cells (21.12 ± 0.9% vs. 9.7 ± 1.2% annexinV+, $P < 0.0001$) compared to Tf-NP-siRNAsc treatment at 96 hours (Supplementary Figure S4C). In addition, after 24 hours of priming cells with *KRAS*, *NRAS* and *MAPK1* siRNA treatment, DNR was added to treat the cells for another 72 hours. We observed that similar to *miR-181a* treatment the *KRAS*, *NRAS* and *MAPK1* siRNA treatment enhanced the apoptotic effect of DNR in both cell lines, MV4-11 (siRNA - > 0.01 μM DNR: 78.5 ± 2.7% vs. scramble - > 0.01 μM DNR: 21.3 ± 10.2% annexinV+, $P = 0.0009$) and OCI-AML3 (siRNA - > 0.04 μM DNR: 56.2 ± 1.3% vs. scramble - > 0.04 μM DNR: 38.6 ± 0.4% annexinV+, $P < 0.0001$; Supplementary Figure S4C).

## Lentiviral induced KRAS, NRAS or MAPK1 expression rescues anti-proliferative Tf-NP-*miR-181a* treatment effects

Lentiviruses containing KRAS, NRAS and MAPK1 were produced as described above. OCI-AML3 cells were infected with lentivirus to overexpress KRAS, NRAS and MAPK1 respectively. Lentiviral-miR-scramble (Lenti-sc) infected cells were used as control in this study. The overexpression of KRAS, NRAS and MAPK1 were confirmed by western blot (Supplementary Figure S5A–S5C) compared to Lenti-sc cell. Then for each group, lenti-sc, KRAS, NRAS and MAPK1, cells were treated with either Tf-NP-sc or Tf-NP-miR-181a (final miR concentration at 10 nM). Cells were harvested and counted at 24-hour intervals using a Bio-Rad TC20 Automated Cell Counter (Bio-Rad, Berkeley, CA). Each sample was run in triplicate. Overexpression KRAS, NRAS or MAPK1 was able to rescue the Tf-NP-miR-181a treatment induced reduced proliferation (Supplementary Figure S5A–S5C). Student *t*-test was used for statistic analysis and *P*-values between each comparison were showed in Supplementary Table S3. In general, the overexpression of NRAS and MAPK1 in OCI-AML3 cells led to slightly more proliferation of the cells compared to lenti-sc infected cells whereas KRAS overexpression had the significant impact of accelerating the growth of cells. After the treatment of Tf-NP-*miR-181a*, KRAS, NRAS and MAPK1 overexpressed cells showed more proliferation comparing to lenti-sc infected cells, which indicated that overexpression of these three genes rescued the cellular phenotype caused by elevated level of *miR-181a* by nanoparticles (*P*-value: lenti-sc+miR-181a vs. lenti-KRAS+miR-181a: 0.004; lenti-sc+miR-181a vs. lenti-NRAS+miR-181a: 0.011; lenti-sc+miR-181a vs. lenti-MAPK1+miR-181a: 0.091). The growth of the cells that overexpressed KRAS, NRAS and MAPK1 then treated by Tf-NP-miR-181a were higher or similar to lenti-sc infected cells treated with Tf-NP-sc.

## Leukemic cell isolation and sorting

The female NOD/SCID mice were engrafted with MV4-11 cells for each treatment group and sacrificed after 8 doses treatment as previously described. Bone marrow cells were collected from mice femurs, and splenocytes were isolated from smashed spleens. The cell suspension was incubated with RBC lysis buffer (eBioscience, San Diego, CA) for 10-min on ice and washed with PBS. To collect human leukemic cell from mouse tissue, the cell suspension was further stained with anti-human CD45 (hCD45) –PE (eBioscience, San Diego, CA) and sorted by FACS Aria II Flow Cytometer (BD, Franklin Lakes, NJ, USA). The hCD45+ population was identified as leukemic cell by the FCSexpress software (De Novo Software, Glendale, CA).

## Supplementary Table S1: PCR primer

| Primer | Sequence |
|---|---|
| *miR-181a-1* Forward | gcgtgctagcCCGATCCTTTTCTCTCATAC |
| *miR-181a-1* Reverse | gcgtggatccGATGGAATATCTGTTGATTG |
| *KRAS* S1 Forward | gtgcgaattcactaatttcagttgagaccttc |
| *KRAS* S1 Reverse | gtgcgaattctaatagtttccattgccttg |
| *KRAS* S1 mutation Forward | catccctgatgTTCgtaaagttac |
| *KRAS* S1 mutation Reverse | gtaactttacGAAcatcagggatg |
| *KRAS* S2 Forward | gtgcgaattccacagagctaactgggttac |
| *KRAS* S2 Reverse | gtgcgaattcgatatgaccaacattcctaggtc |
| *KRAS* S2 mutation Forward | catgtttacctggaaCCCattttaac |
| *KRAS* S2 mutation Reverse | gttaaaatGGGttccaggtaaacatg |
| *NRAS* Forward | gtgcgaattctgagtctatcctagtcttca |
| *NRAS* Reverse | gtgcgaattctttcatctttctcctgggaa |
| *NRAS* mutation Forward | atccttatgcatgaaatgCCCgtctgag |
| *NRAS* mutation Reverse | ctcagacGGGcatttcatgcataaggat |
| *MAPK1* Forward | gtgcgaattcgtactgttggtgccttcttggtat |
| *MAPK1* Reverse | gtgcgaattccaggtgccataaacattcaaataatccatc |
| *MAPK1* S1 mutation Forward | ggaagattttattaagaatctgTCAAtttattc |
| *MAPK1* S1 mutation Reverse | gaataaaTTGAcagattcttaataaaatcttcc |
| *MAPK1* S2 mutation Reverse | gtgcgaattccaggtgccataaaTTGAcaaataatccatc |
| *KRAS* clone Forward | aaattaaattgctagcATGACTGAATATAAACTTGTGGTAGTTGG |
| *KRAS* clone Reverse | aaattaaattggatccTTACATAATTACACACTTTGTCTTTGACTTCTT |
| *NRAS* clone Forward | gtgcggatccGTGTGAAATGACTGAGTAC |
| *NRAS* clone Reverse | gtacggatccGTATCTTGTTACATCACCAC |
| *MAPK1* clone Forward | ttaaattaaaTCTAGAATGGCGGCGGCGGCGGC |
| *MAPK1* clone Reverse | ttaaattaaaGCTAGCTTAAGATCTGTATCCTGGCTGGAATCTAGCAGTCTCTTC |

## Supplementary Table S2: Characteristics of AML patients

| | Karyotype | *FLT3-ITD* | *NPM1* | *CEBPA* |
|---|---|---|---|---|
| Pat #1 | 46, XX | present | mutated | wild-type |
| Pat #2 | 46, XX | present | wild-type | wild-type |
| Pat #3 | 46, XX | present | wild-type | wild-type |
| Pat #4 | 46, XX | present | wild-type | mutated |
| Pat #5 | 46,XX | absent | mutated | wild-type |
| Pat #6 | 46, XY, inv(16)(p13q22)/46, | absent | wild-type | wild-type |
| Pat #7 | 46, XY, del(11)(p13p15)/46, | absent | wild-type | wild-type |
| Pat #8 | NA | present | mutated | wild-type |
| Pat #9 | complex karyotype | absent | wild-type | wild-type |

## Supplementary Table S3: Statistic analysis

| *P*- value | Lenti-sc +miR-sc | Lenti-sc +miR-181a | Lenti-KRAS +miR-sc | Lenti-NRAS +miR-sc | Lenti-MAPK1 +miR-sc |
|---|---|---|---|---|---|
| **Lenti-sc +miR-sc** | 1 | | | | |
| **Lenti-sc +miR-181a** | 0.024 | 1 | | | |
| **Lenti-KRAS +miR-sc** | 0.006 | 0.002 | 1 | | |
| **Lenti-KRAS +miR-181a** | 0.050 | 0.004 | 0.158 | | |
| **Lenti-NRAS +miR-sc** | 0.326 | 0.029 | | 1 | |
| **Lenti-NRAS +miR-181a** | 0.259 | 0.011 | | 0.833 | |
| **Lenti-MAPK1 +miR-sc** | 0.060 | 0.008 | | | 1 |
| **Lenti-MAPK1 +miR-181a** | 0.178 | 0.091 | | | 0.051 |

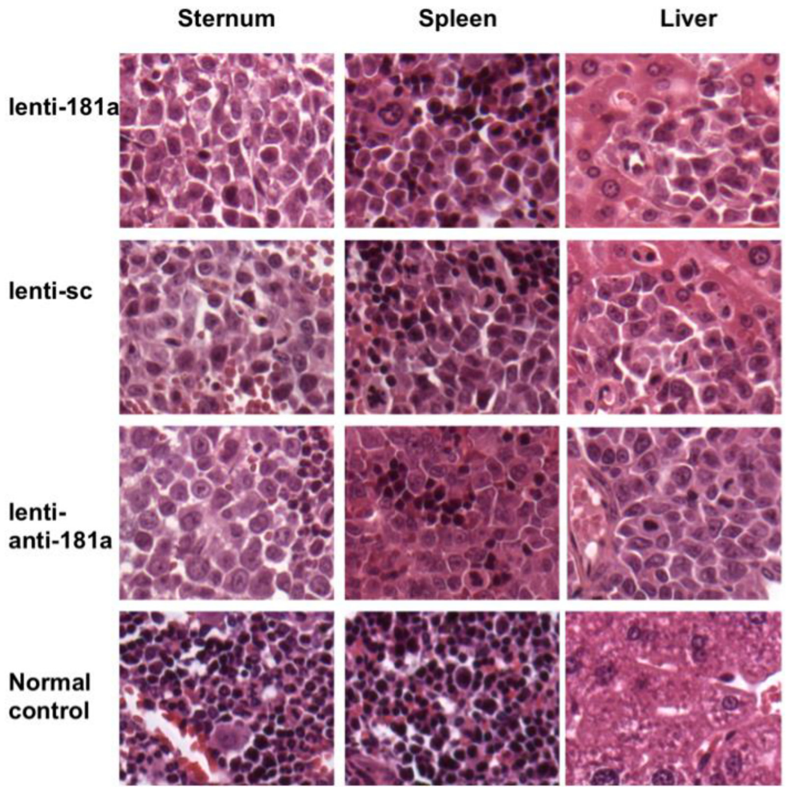


**Supplementary Figure S1: H&E staining of sections from sternum, spleen and liver sections of virally transduced-MV4-11 engrafted mice.**

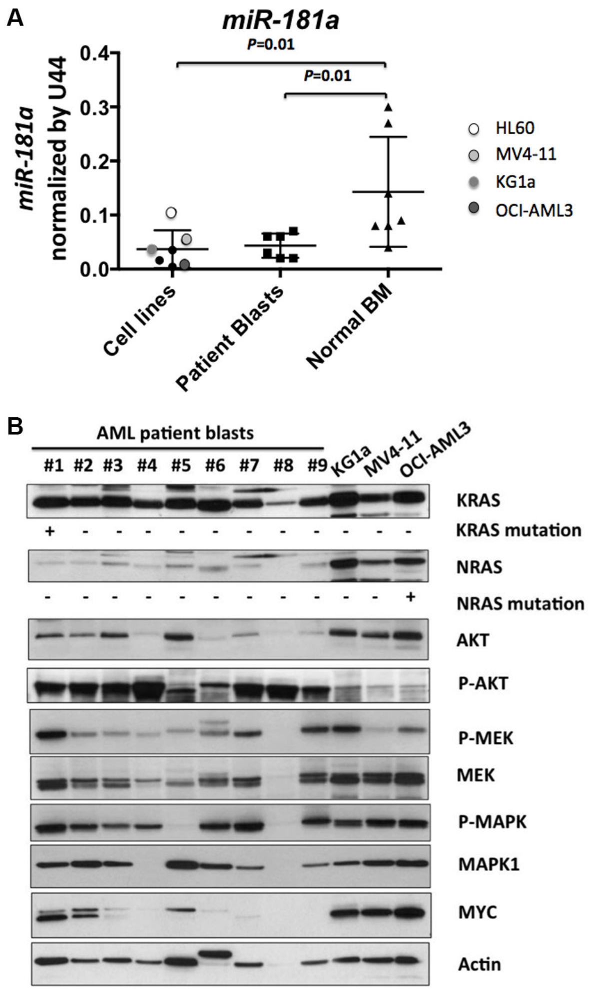


**Supplementary Figure S2:** (**A**) *miR-181a* expression levels comparing AML cell lines, primary AML patient blasts and bone marrow cells of healthy donors. (**B**) NRAS, KRAS, p-MEK, MEK, p-AKT, AKT, p-MAPK, MAPK1, and MYC protein expression in blasts cells from nine AML patients and KG1a, OCI-AML3 and MV4-11 cells.

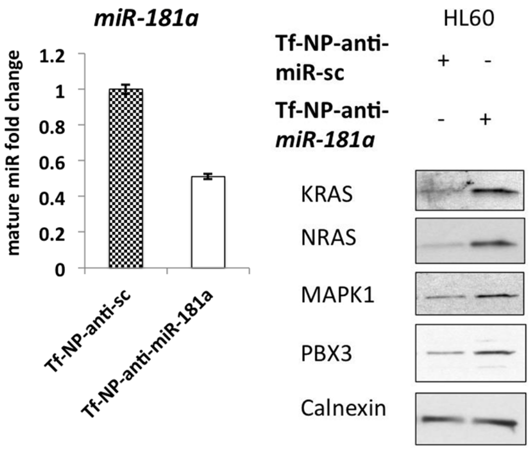


**Supplementary Figure S3: Downregulation of *miR-181a* by Tf-NP-anti-*miR-181a* and upregulation of *miR-181a*'s targets, KRAS, NRAS MAPK1 and PBX3.**

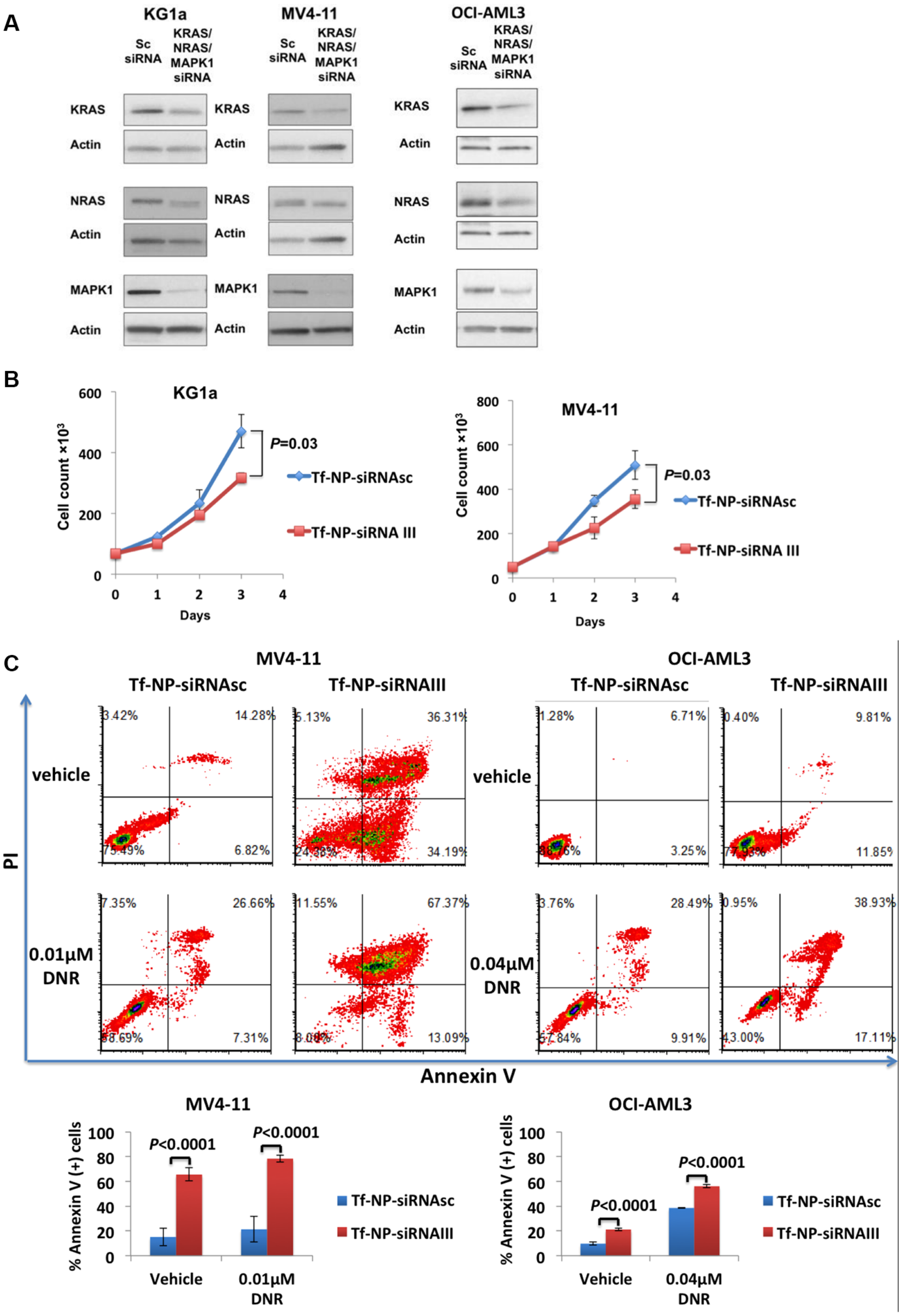


**Supplementary Figure S4:** (**A**) Downregulation of KRAS, NRAS and MAPK1 proteins following their respective siRNA treatments in KG1a, MV4-11 and OCI-AML3. Three siRNAs against KRAS, NRAS and MAPK1 (siRNAIII) were equally mixed and encapsulated into Tf-NP. Two AML cell lines, KG1a and MV4-11 were treated with Tf-NP-siRNAIII at a concentration of 60 nM for each KRAS, NRAS and MAPK1 siRNA. 180 nM of scramble siRNA contained Tf-NP as a control. (**B**) Growth curve of KG1a and MV4-11 cell lines after KRAS/NRAS/MAPK1 siRNAs treatments compared with scramble siRNA treatment. (**C**) Annexin V assays in MV4-11 and OCI-AML3 cells treated with Tf-NP-siRNAIII, Tf-NP-scsiRNA in the presence or absence of daunorubicin (DNR, 0.01 µM for MV4-11, 0.04 µM for OCI-AML3. DNR was added 24 hours after priming cells with Tf-NP-*miR-181a* for 72 hours.

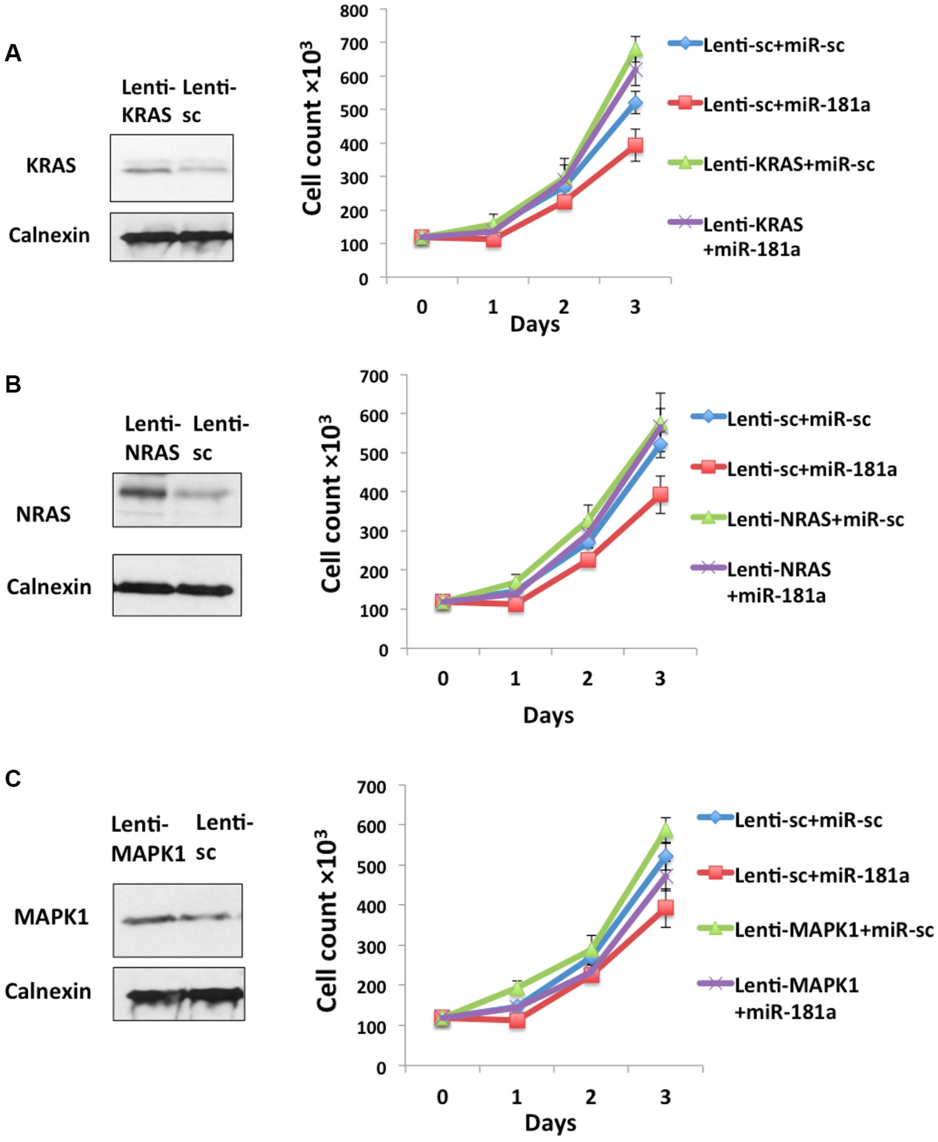


**Supplementary Figure S5: KRAS (A), NRAS (B) and MAPK1 (C) were overexpressed by lenti-virus respectively in OCI-AML3 cells.** Then these cells along with control cells were treated with Tf-NP-sc or Tf-NP-*miR-181a* for cell growth study.

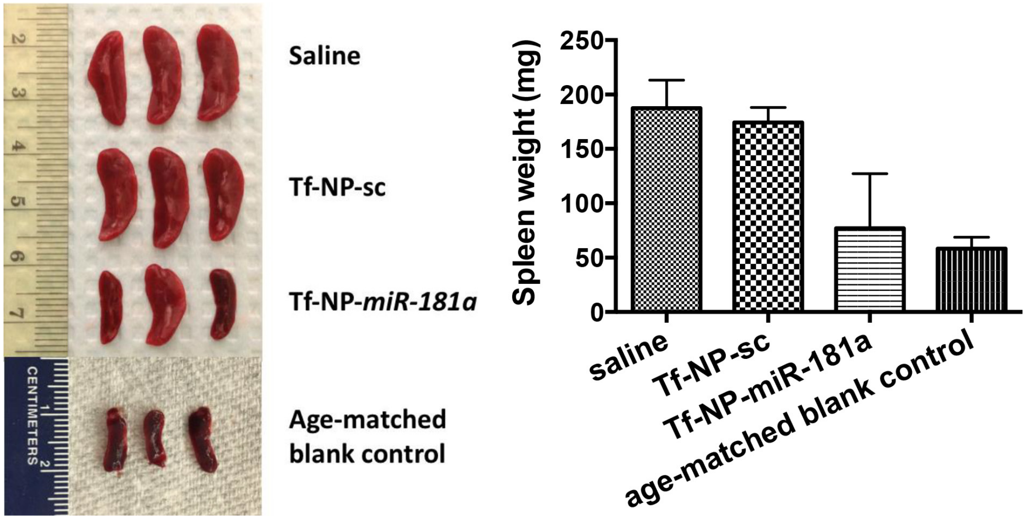


**Supplementary Figure S6: Spleens (left) and spleen weights (right) from mice sacrificed after 8 doses of treatment from each group, saline, Tf-NP-sc, Tf-NP-*miR-181a* ($n$ = 3) and age-matched blank controls ($n$ = 3)**.
